# Novel coronavirus 2019-nCoV (COVID-19): early estimation of epidemiological parameters and epidemic size estimates

**DOI:** 10.1098/rstb.2020.0265

**Published:** 2021-07-19

**Authors:** Jonathan M. Read, Jessica R. E. Bridgen, Derek A. T. Cummings, Antonia Ho, Chris P. Jewell

**Affiliations:** ^1^ Centre for Health Informatics, Computing and Statistics, Lancaster Medical School, Lancaster University, Lancaster LA1 4AT, UK; ^2^ Department of Biology and Emerging Pathogens Institute, University of Florida, Gainesville, FL 32611, USA; ^3^ Medical Research Council - University of Glasgow Centre for Virus Research, Glasgow G61 1QH, UK

**Keywords:** SARS-CoV-2, transmission model, pandemic, ascertainment rate, China, international travel

## Abstract

Since it was first identified, the epidemic scale of the recently emerged novel coronavirus (2019-nCoV) in Wuhan, China, has increased rapidly, with cases arising across China and other countries and regions. Using a transmission model, we estimate a basic reproductive number of 3.11 (95% CI, 2.39–4.13), indicating that 58–76% of transmissions must be prevented to stop increasing. We also estimate a case ascertainment rate in Wuhan of 5.0% (95% CI, 3.6–7.4). The true size of the epidemic may be significantly greater than the published case counts suggest, with our model estimating 21 022 (prediction interval, 11 090–33 490) total infections in Wuhan between 1 and 22 January. We discuss our findings in the light of more recent information.

This article is part of the theme issue ‘Modelling that shaped the early COVID-19 pandemic response in the UK’.

## 1. Introduction

A novel betacoronavirus (SARS-CoV-2) [1] was first identified from a cluster of atypical pneumonia cases in Wuhan, Hubei Province, China, on 31 December 2019. Most initial cases had epidemiological links with a live animal market, suggesting a possible zoonotic origin [2]. Over the following six weeks, cases spread to other Chinese provinces. As of 17 February 2020, 09.00 GMT, there are over 50 000 confirmed cases—the majority in mainland China—and more than 600 cases reported in 25 other countries [3]. Infections in family clusters [4,5] and in healthcare workers confirm the occurrence of human-to-human transmission. Furthermore, recent case clusters in Germany [6], France [7] and on a cruise ship in Japan [2] suggest that SARS-CoV-2 is highly transmissible. Emerging data suggest that coronavirus disease 2019 (COVID-19) causes a spectrum of clinical severity, from mild upper respiratory tract illness to severe pneumonia, with a small proportion developing acute respiratory distress syndrome (ARDS), septic shock, multi-organ failure and death [4,5,8,9]. However, the proportion of those infected that have mild symptoms and do not seek medical care is unclear, since surveillance is likely biased towards severe disease.

**Figure 1.**
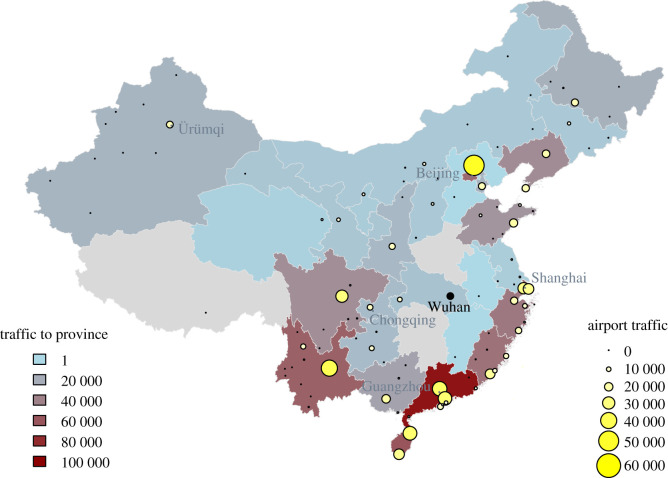
Connectivity of Wuhan to other cities and provinces in mainland China, based on total commercial airline traffic from Wuhan in January 2017. Traffic is based on the number of departing bookings. (Online version in colour.)

Wuhan is a city of more than 11 million residents and is connected to other cities in China via high-speed railway and frequent commercial airline flights. There were 670 417 airline passenger bookings departing Wuhan made during January 2017, the top destinations being Shanghai (53 214 bookings), Beijing (51 066 bookings) and Kunming (40 120 bookings) [10] (figure 1). While the majority of air travel departing Wuhan is domestic (87.2% of bookings, January 2017), Wuhan is connected internationally through both direct and indirect flights [11]. The outbreak comes at a time when there is a substantial increase in travel volume within as well as in and out of China around the Lunar New Year on 25 January 2019. Over 3 billion passenger journeys were predicted for the period between 10 January and 18 February [12]. In an effort to contain the outbreak, travel restrictions were imposed in Wuhan from 23 January, and have since expanded to 12 other cities, and large social gatherings cancelled [13].

Here, we fitted a mathematical model of transmission within Wuhan and other Chinese cities to early reported numbers of confirmed cases within cities in China and in other countries or regions. We inferred the rate of underreporting in Wuhan to estimate the possible size of the outbreak in Wuhan, as well as key epidemiological parameters including the basic reproductive ratio and infectious period.

## 2. Methods

### (a) Transmission model

We fitted a deterministic SEIR (Susceptible-Exposed-Infectious-Removed) metapopulation transmission model of infection within and between major Chinese cities to the daily number of confirmed cases of COVID-19 in Chinese cities and cases reported in other countries/regions, using an assumption of Poisson-distributed daily case counts. We modelled the period from 1 January 2020, when local authorities closed the wet market implicated as the zoonotic source of human infection [14], up to and including 22 January 2020. We only considered human-to-human transmission in our model and made the assumption that following the closure of the market on 1 January, no further zoonotic infection contributed to epidemic dynamics. Further description of the mathematical model can be found in the electronic supplementary material.

We modelled transmission of infection between cities in China using daily-adjusted rates of travel estimated from monthly aggregated full itinerary passenger booking data for January 2017, accessed from OAG Traffic Analyser database [10]. We also modelled the expected importation of infection into other countries or regions outside of mainland China, using the same data. We made the assumption that travellers are drawn randomly from the origin population, and, therefore, the prevalence of infection among travellers is the same as the prevalence in the population travellers are starting from.

We estimated the transmission rate and the removal rate (the inverse of the effective infectious period) through fitting the model to daily case numbers reported within the modelled cities in China and reported by other countries/regions. We assumed that the latent period was 4 days, based on an estimate of the incubation period of SARS, a related coronavirus [15]. This is similar to the estimate of 4.4 days from the initial characterization of COVID-19 cases. We made the assumption that the latent period approximates to the incubation period. We also estimated the case ascertainment ratio (CAR) within Wuhan, and the initial number of human infections present in Wuhan when the market was closed. We assumed that the case ascertainment is 100% in other cities in China, as well as in other countries/regions; we note that this assumption may be an overestimate of the CAR in other locations [16].

### (b) Parameter estimation

Daily numbers of newly confirmed cases in Chinese cities and other countries/regions reported up to and including 22 January 2020 were used for fitting; data were collated from public reports. For model validation, we compared model out-of-sample predictions for the period 23–29 January, using data collated in the same way. From 23 January, cases for Wuhan and other locations within Hubei were only reported at the aggregate province level. Fitting was achieved by treating the ordinary differential equations (ODE) system as representing the mean number of new cases per day in our study period, and assuming that the observed number of new cases was (approx.) Poisson distributed around this mean. Given the model and data, parameter inference was achieved by maximum-likelihood estimation using the Nelder–Mead optimization as implemented in the *optim*() function in the R statistical language [17] (see https://github.com/chrism0dwk/wuhan/tree/v0.3 for R code, case data and prepared datafiles).

Uncertainty in the parameter estimates was explored using parametric bootstrap according to the following procedure. Firstly, 10 000 Monte Carlo simulations from the model (ODE and Poisson noise) were generated using the maximum likelihood estimates of the parameters. Each simulated dataset was then re-fitted to the model to construct a joint sampling distribution of the parameters, and 95% confidence estimated as the lower 2.5% and upper 97.5% quantiles. The ODE system (without Poisson noise) was run over this sampling distribution to generate 95% confidence intervals around the predicted mean epidemic trajectory.

### (c) Doubling time calculation

We calculate the doubling time of an epidemic using the observed cumulative epidemic size, *q*, at two time points, *t*1 and *t*2. The epidemic doubling time, Td, is given by

$$\mathrm{Td}=(t2-t1)\left(\frac{\log(2)}{\log(q_{t2}/q_{t1})}\right).$$

## 3. Results

### (a) Epidemiological parameter estimates

We estimated the transmission rate within Wuhan, *β*, to be 1.94 days^−1^ (95% CI, 1.25–6.71), while we found the infectious period to be 1.61 days (95% CI, 0.35–3.23). We calculated the basic reproductive number, *R*_0_, of the infection to be 3.11 (95% CI, 2.39–4.13), comparable to the range for SARS estimated from outbreaks during the 2003 epidemic [18,19], as well as other early estimates for COVID-19 [20–24]. We highlight that this number is highly uncertain and that a large range of parameters are consistent with the data given the assumptions of our model. This estimate reflects both the dynamics of transmission and, potentially, the dynamics of case reporting, where increases in reporting rate over time could potentially inflate our estimate. This estimate of *R*_0_ is significantly greater than 1, the epidemic threshold, suggesting a concerted effort is required to control the outbreak, requiring between 58% and 76% of transmission to be averted to control the epidemic.

We estimated that the average CAR in Wuhan between 1 and 22 January was 5.0% (95% CI, 3.6%–7.4%), reflecting the difficulty in identifying cases of a novel pathogen. Given the generally good level of access to healthcare in China, this also suggests that the majority of infections may be of mild illness and insufficiently serious for individuals to seek treatment. However, it is worth noting that a number of identified cases have died [25] and that uncertainty in the case fatality ratio remains. Also, asymptomatic infection has been reported for COVID-19 [4]. Finally, we also estimated the size of the epidemic in Wuhan at the time of the market closure (1 January) to be 15 individuals (95% CI, 5–37).

**Figure 2.**
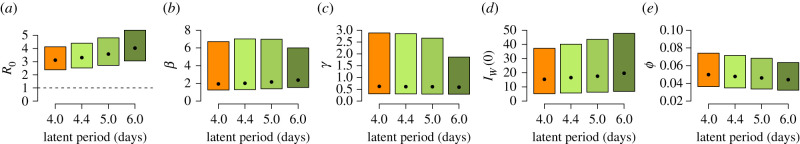
Sensitivity of parameter estimates to the assumed latent period (1/*α*) value. Boxes represent the 2.5% and 97.5% quantiles and black dots the 50% quantile. (*a*) Basic reproductive number, (*b*) transmission rate, (*c*) recovery rate, (*d*) infections, 1 January, and (*e*) case ascertainment ratio. (Online version in colour.)

Our estimates of epidemiological parameters are sensitive to our assumption regarding the length of the latent period (figure 2). Early epidemiological investigations suggest a duration between 3 and 6 days [4]; should the latent period be longer than the 4 days we assume, our *R*_0_ estimates would be higher and the estimated CAR slightly lower (figure 2). If cases were reported with increasing efficiency or the timing of cases is inconsistent with the timing assumed here (i.e. throughout the outbreak, the length of time between infection and reporting in surveillance data is declining), this may tend to decrease our estimate of the reproductive number.

### (b) Epidemic size estimates

Using our parametrized transmission model, we simulated the impact of an ongoing outbreak in Wuhan to seed infections and outbreaks in other cities of China, and to generate infection in travellers to other countries/regions, through airline travel originating in China. We stress that these projections make strong assumptions: that no control interventions are instigated; that the key epidemiological variables driving epidemic dynamics remain constant; that travel behaviour within China and to other countries/regions continues as per our mobility estimates; finally, we only consider travel by air and do not include land transportation, particularly via the rail network within China.

We estimated that on 22 January, in Wuhan, there were currently 14 464 infected individuals (prediction interval, 6510–25 095), and a total 21 022 infections (prediction interval, 11 090–33 490) since the start of the year. We also estimate there were 24 currently infected individuals (prediction interval, 19–30) in other locations of China on this date. For comparative purposes, we estimate the total number of infections in Wuhan from 1 January to 18 January inclusive to have been 6733 (prediction interval, 3500–10 914). This estimate of the total infections is comparable to other published estimates based on travel data and reported cases identified outside of China (estimated between 1700 and 7800) [26], and highlights our estimated low CAR, the rapid growth of the epidemic and uncertainty in model predictions.

**Figure 3.**
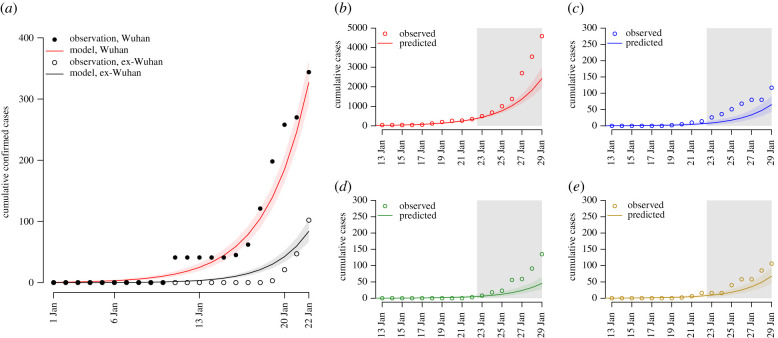
(*a*) Comparison of observed cases and predicted cumulative confirmed cases in Wuhan for the period 1–24 January. Out-of-scope epidemic predictions of cumulative confirmed cases for (*b*) Hubei, (*c*) Beijing, (*d*) Guangzhou and (*e*) Shanghai up to 29 January. Grey region denotes the prediction period; 95% confidence intervals around the mean epidemic trajectories are denoted by coloured areas.

**Figure 4.**
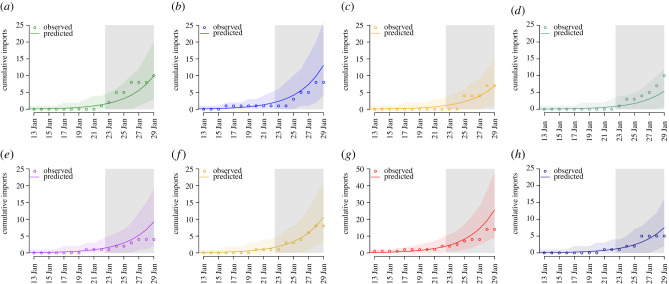
Out-of-scope predictions of cumulative confirmed cases in selected countries/regions up to 29 January. Grey region denotes the prediction period; 95% confidence intervals around the mean epidemic trajectories are denoted by coloured areas. (*a*) Hong Kong SAR, (*b*) Japan, (*c*) Malaysia, (*d*) Singapore, (*e*) South Korea, (*f*) Taiwan, (*g*) Thailand and (*h*) USA. (Online version in colour.)

From 23 January, large-scale movement restrictions were implemented in Wuhan and across Hubei province in an effort to contain the spread of the virus. For the period 23–29 January, our model underestimated the growth of epidemics within Hubei and other Chinese cities (figure 3*b–e*), while our predictions for exportations to other countries/regions were reasonable (figure 4). While this could reflect an increase in the transmission rate, it may also be due to accelerated case detection, an increase in testing capacity, changes in case definition or reflect delays in reporting cases within China.

Our model predicts that on 29 January, the epidemic in Wuhan will be substantially larger, with 594 cases expected to be detected on that day in Wuhan (prediction interval, 446–788) and 105 077 currently infected (prediction interval, 46 635–185 412) (figure 3 and table 1). If transmission has reduced, either through control or spontaneous public response to the epidemic, this will be a gross overestimate, though it may be useful to help gauge the effectiveness of interventions.

**Table 1.** Predicted epidemic sizes (number of detected cases) in selected Chinese cities and predicted imports to other countries/regions on 29 January 2020, assuming no change in transmissibility or ascertainment ratio. Assumption 1: CAR in all cities excluding Wuhan is 100%. Assumption 2: CAR in all Chinese cities is that estimated for Wuhan (5.0%).

		predicted detected cases on 29 Jan 2020									
		assumption 1			assumption 2				number of infected importations (days^−1^)		
rank	city	mean	2.5% quantile	97.5% quantile	country/territories	mean	2.5% quantile	97.5% quantile	mean	2.5% quantile	97.5% quantile
1	Wuhan	594	446	788	594	446	788	Thailand	6.3	2.8	11.1
2	Shanghai	19	13	26	1	1	2	Japan	3.2	1.5	5.7
3	Beijing	18	13	25	1	1	2	Taiwan	2.6	1.2	4.6
4	Kunming	14	10	19	1	0	1	Hong Kong SAR	2.5	1.1	4.3
5	Guangzhou	13	9	17	1	0	1	South Korea	2.3	1.0	4.0
6	Haikou	12	8	16	1	0	1	USA	1.8	0.8	3.3
7	Shenzhen	12	8	16	1	0	1	Malaysia	1.7	0.8	3.0
8	Chengdu	11	8	15	1	0	1	Singapore	1.3	0.6	2.3
9	Sanya	9	6	12	0	0	1	Australia	1.2	0.5	2.2
10	Xiamen	8	6	11	0	0	1	Viet Nam	1.1	0.5	2.0
11	Nanning	7	5	10	0	0	1	Indonesia	1.1	0.5	2.0
12	Qingdao	7	5	9	0	0	1	Macau SAR	1.1	0.5	1.9
13	Shenyang	6	4	9	0	0	1	Cambodia	0.6	0.3	1.0
14	Hangzhou	6	4	8	0	0	0	UK	0.5	0.2	0.8
15	Dalian	6	4	8	0	0	0	Canada	0.4	0.2	0.8
16	Harbin	6	4	8	0	0	0	France	0.4	0.2	0.7
17	Tianjin	6	4	8	0	0	0	United Arab Emirates	0.4	0.2	0.7
18	Ürümqi	5	4	7	0	0	0	Philippines	0.4	0.2	0.6
19	Wenzhou	5	4	7	0	0	0	Germany	0.3	0.1	0.5
20	Xi'an/Xianyang	5	4	7	0	0	0	India	0.2	0.1	0.4

The model predicts infected travellers to other Chinese cities will initiate outbreaks in those cities, the largest on 29 January being in Shanghai, Beijing, Guangzhou, Shenzhen, Chengdu and Kunming (figure 3 and table 1). Our model predicts the total number of infected individuals in locations elsewhere in China to be 237 (prediction interval, 167–324) on 29 January.

Finally, the model predicts an elevated risk of importations into other countries/regions, most notably to Thailand, Japan, South Korea, Taiwan, Hong Kong SAR, USA, Singapore, Malaysia, Australia and Viet Nam (figure 3 and table 1). Again, these predictions assume no change in the transmission of the virus within China through control or other responses to the epidemic, and likely underestimate the potential importation rate to regions with ground transportation from China, in particular Hong Kong.

## 4. Comparison of estimates to other reports

Our estimates of *R*_0_ are broadly consistent with early estimates from other groups: 2.5 (95% CI 2.2–2.9) for peer-reviewed studies and 3.6 for pre-prints (95% CI 2.7–4.5) [27]; 2.6 (uncertainty range 1.5–3.5) [28]; 2.92 (95% CI 2.28, 3.67) [29]; 2.2 (90% interval: 1.4–3.8) [30]. Sources of discrepancies may be due to model differences and differences in the contribution of specific types of data to our estimates. We believe that our estimates are slightly elevated compared to others due to the inclusion of cases from other locations within China other than Wuhan. However, it is important to note that our point estimate is consistent with all others' uncertainty intervals, all indicating sustained growth of cases.

## 5. Comparison of transmissibility with SARS and MERS

Our estimates of the basic reproductive number for this novel coronavirus are comparable to most estimates reported for SARS and MERS-CoV, but similar to some estimates from subsets of data in the early period of SARS. For the SARS coronavirus, estimates of the mean reproductive number ranged from 1.1 to 4.2 with most estimates between 2 and 3 [31]. These estimates represent a range of methods and settings. Some estimates come from data that mix time periods before and after control. Estimates of *R*_0_ also varied based on assumed serial intervals (e.g. [18] estimated *R*_0_ ranging from 2.2 to 3.6 for serial intervals of 8–12 days). Another study [31] reviewed sources of variation in basic reproductive numbers of SARS and noted that in those locations in which outbreaks occurred, *R*_0_ was approximately 3. Estimates from MERS-CoV were uniformly lower, with estimates from Saudi Arabia having a mean of less than 1 (approx. 0.5) but exhibited large temporal variability with increases in some periods of time, particularly in healthcare settings [32].

A comparison of the efficiency of transmission in this outbreak and in SARS outbreaks can be seen as well in simple comparisons of doubling times in each outbreak. In SARS, doubling times varied from 4.6 to 14.2 days depending on setting: doubling time, Td = 6.0 (1358 over 63 days, Singapore), Td = 4.6 (425 over 41 days, Hong Kong), Td = 14.2 (7919 over 185 days, overall) [18]. Using confirmed case information (41 reported 14 January; 291 reported 24.00 on 20 January; 1975 reported 24.00 on 25 January) [33], we find doubling times of 2.1, 1.8 and 2.0 days. If the outbreak has been ongoing for a longer period of time, this would increase the estimated doubling time. These doubling time estimates, similar to our estimates of *R*_0_, are susceptible to bias due to the dynamics of case reporting, with bunching of identified cases (due to temporally clustered recognition of cases) tending to bias our estimate towards lower doubling times. We note our estimates of the doubling time in this outbreak are short compared to estimates from the SARS outbreak in Hong Kong [18].

## 6. Limitations

Our model necessarily makes a number of assumptions. Our estimates of the basic reproductive number of this novel coronavirus are tied to the specific time period and data analysed here, and this measure may change substantially over the course of this outbreak and as additional data arrive. Additionally, the spatial component of our model is dependent upon only airline travel; the model does not include rail and road transportation, so we may underestimate local connectivity and the connectivity of Wuhan to other locations. We also do not attempt to account for any implementation of control, nor any dynamic changes of factors that may influence transmission (such as spontaneous social distancing), nor changes in surveillance and reporting effort. Our choice of modelling approach may also lead to unreliability in the precision of our estimated model transmission parameters [34]. However, our approach used ‘raw’ counts of cases to fit the model, not cumulative case information, and as such, our point estimates would not be biased [34]. Finally, we made a pragmatic assumption that all infections in Chinese cities excluding Wuhan and in international destinations were identified (CAR is 100%). Significant prevalence of asymptomatic and pre-symptomatic infections (particularly where border screening of travellers relies on symptomatic detection) would mean the number of cases outside of Wuhan and in other countries we use for model fitting are underestimates, resulting in an underestimate of the inter-city and international transmission rate. This may be partially offset by non-Wuhan locations generating their own cases (inflating the number of infections relative to what we would expect if Wuhan were the only case generator). Our transmission rate estimates would be robust if the ratio of asymptomatic infection to symptomatic of these occult infections were unbiased (e.g. occult infection prevalence was identical in Guangzhou, Beijing and among international travellers) and there was uniform diagnostic ability.

Earlier novel coronavirus (SARS and MERS-CoV) outbreaks found evidence for substantial heterogeneity in reproductive numbers between individuals [31,32,35]. In our analysis, we assume that there is little heterogeneity in reproductive numbers and this assumption may change our estimated reproductive number. Additionally, *R*_0_ estimates tend to be reduced as case information accumulates, though control measures may also be introduced during these periods. As is true for any modelling analysis of surveillance data, our estimate of *R*_0_ may also reflect the dynamics of surveillance effort and reporting rather than just the dynamics of the epidemic.

A key uncertainty of this outbreak is when it started. We have chosen to model transmission from 1 January onwards. Surveillance in China and elsewhere only started once the outbreak was identified in Wuhan. Had the outbreak started much earlier, and both within China and international infectious exports occurred before January and in early January (while surveillance was ramping up), our estimates of the reproductive number would mostly decrease.

A threat to the accuracy of these projections is if a substantial proportion of infection has been due to multiple exposures to animals that have been curtailed in some way. These data may also represent a period of high transmission (due to favourable seasonal conditions, stochastic variation or selection bias in detecting large clusters of transmission) that will not be sustained over long periods of time.

## 7. Summary

We are still in the early days of this outbreak and there is much uncertainty in both the scale of the outbreak and key epidemiological information regarding transmission. However, the rapidity of the growth of cases since the recognition of the outbreak is much greater than that observed in outbreaks of either SARS or MERS-CoV. This is consistent with our broadly higher estimates of the reproductive number for this outbreak compared to these other emergent coronaviruses, suggesting that containment or control of this pathogen may be substantially more difficult.

## 8. In context

This work was conducted, written and uploaded to an open-access preprint repository in January 2020; figures 3 and 4 were revised shortly afterwards in early February 2020. As such, it provided early estimates of important epidemiological characteristics of SARS-CoV-2 and contributed to the evidence that the novel coronavirus had pandemic potential. Here, we reflect on our modelling approach and the findings of the research in the light of the considerable research conducted on SARS-CoV-2 since.

### (a) Methodological approach

The modelling approach and inference methodology used in this study was necessarily simple: ODE are quick to implement, with the formulation of the likelihood as Poisson distributed noise around the ODE system being a pragmatic way of accounting for stochastic variation in daily case detections. Many approaches to modelling SARS-CoV-2 have continued with these assumptions. However, even though case reporting will be subject to measurement error, this approach ignores the fact that epidemics are intrinsically stochastic—the ODE approach does not allow for stochastic jumps in the mean epidemic trajectory. This is particularly problematic for models trained on datasets with low numbers of cases, where stochastic variation in the epidemic process dominates the overall epidemic dynamics. In such cases, the variation in the case time series will be attributed to the overdispersion, leading to non-identifiability of the model parameters of interest. Work on statistical inference methods for stochastic methodology is an ongoing topic of research, and is a much needed area of future methodological development to ensure that rapid, accurate and fine-scale calibration of complex models is feasible in the event of an outbreak.

### (b) Assumptions regarding pathogen epidemiology

Transmission modelling conducted during the early stages of any outbreak of a novel pathogen necessarily makes a number of strong assumptions regarding the epidemiology of the pathogen, and there are several in this work which may now be reassessed in the light of more recent studies. A key assumption made was the length of the latent period. In January 2020, no epidemiological studies from China had been published that described this interval. Through analogy, we assumed a similar distribution to that observed as the incubation period for the related virus, MERS-CoV. A study published in March 2020 found the incubation period for SARS-CoV-2 in early Wuhan cases was 5.2 days (95% CI, 4.1–7.0) [22], while Linton *et al*. [36] estimated the incubation period to be between 3.5 and 5.7 days (they present a range of estimates using different methods). Further, Lauer *et al*. [37] estimated the period as 5.1 days (95% CI, 4.5–5.8).

By modelling the SEIR infection states as susceptible, latent (infected but not infectious), infectious (whether symptomatic or not) and identified/removed, we effectively incorporated pre-symptomatic transmission and avoided issues around asymptomatic infection, though we did assume that pre-symptomatic and post-symptomatic infected individuals were equally infectious. We also made a strong assumption that case reporting was consistent during the modelled time period. Tsang *et al.* [38] have shown that as the case definition was relaxed (as knowledge accumulated during the early stages of the Wuhan outbreak), a greater number of cases were reported, which could confound estimates of epidemic growth.

### (c) Effective infectious period

Unusually for a transmission model, we were able to jointly estimate both transmission rate and effective infectious period—we attribute this to the constraints imposed by fitting to the spatial–temporal case data. Our estimate of 1.61 days (95% CI, 0.35–3.23) is relatively short compared to the known duration of viral shedding, where the live virus has been isolated from patients up to 9 days since illness onset [39], and compared to one epidemiological study of Chinese cases which estimated the mean infectious period to be 14 days (IQR 11–17.5 days) [40]. Our estimate for the *effective* infectious period also accounts for the effect of treatment or isolation on the period for which individuals may infect others. However, estimates of symptom onset to admission in Wuhan during the modelled period were in the range of 8–14 days [41], suggesting that self-isolation following the onset of illness may have been common.

### (d) Basic reproduction number

Despite the limitations of our approach, in particular the reliance on publicly reported case data, our estimated basic reproduction number for Wuhan compares favourably with estimates for a similar time or in the following months. A study using a similar transmission modelling framework to the one presented here [42] estimated *R*_0_ to be 2.7 (95% CrI, 2.5–2.9). Li *et al.* [22] reported *R*_0_ of 2.2 (95% CI, 1.4–3.9) for Wuhan using data from the same period as our study. A review [43] of early reproduction number estimates based on COVID-19 cases from Wuhan and other locations found estimates to range between 1.9 and 6.5, with the majority between 2.0 and 3.0. Another review [44] found a pooled estimate of *R*_0_ to be 3.0 (95% CI, 2.7–3.4).

### (e) Reflective summary

Our paper demonstrates the utility of relatively simple transmission models for providing a rapid quantitative assessment of disease transmission risk and pandemic potential, given early reports of case incidence of a novel pathogen. Such information may be used to justify the implementation of border disease screening programmes, which may be targeted towards high-risk international transport routes. Despite the reliance on aggregated, publicly reported data, and invoking key assumptions about the natural history of the disease (notably the latent period) albeit informed by that of a closely related virus, our approach provided reasonable estimates of both the basic reproduction number and the likely true scale of the epidemic at the pandemic source. This highlights the usefulness of relatively simple models to capture salient features of an epidemic, despite not incorporating elements now known to be important, such as differential disease severity by age and asymptomatic infection.

## Data accessibility

Case data and R code are available at github respository: https://github.com/chrism0dwk/wuhan/tree/v0.3. Airline flight data are proprietorial and cannot be shared, but is available from OAG (https://www.oag.com/traffic-analyzer). Case data from China cities and from other countries/regions are included as Excel files in the electronic supplementary material. Comments for each cell provide the source of each number. R code for generating figures 2–4 and table 1 is included in the electronic supplementary material.
